# Functional Results after Repair of Large Hiatal Hernia by Use of a Biologic Mesh

**DOI:** 10.3389/fsurg.2016.00016

**Published:** 2016-03-09

**Authors:** Filimon Antonakis, Ferdinand Köckerling, Friedrich Kallinowski

**Affiliations:** ^1^Department of General and Visceral Surgery, Asklepios Klinikum Harburg, Hamburg, Germany; ^2^Department of General, Visceral and Vascular Surgery, Vivantes Klinikum Spandau, Berlin, Germany

**Keywords:** hiatal hernia repair, recurrence, dysphagia, biologic mesh, complications

## Abstract

**Background:**

The aim of this observational study is to analyze the results of patients with large hiatal hernia and upside-down stomach after surgical closure with a biological mesh (Permacol^®^, Covidien, Neustadt an der Donau, Germany). Biological mesh is used to prevent long-term detrimental effects of artificial meshes and to reduce recurrence rates.

**Methods:**

A total of 13 patients with a large hiatal hernia and endothoracic stomach, who underwent surgery between 2010 and 2014, were included. Interviews and upper endoscopy were conducted to determine recurrences, lifestyle restrictions, and current complaints.

**Results:**

After a mean follow-up of 26 ± 18 months (range: 3–58 months), 10 patients (3 men, mean age 73 ± 13, range: 26–81 years) were evaluated. A small recurrent axial hernia was found in one patient postoperatively. Dysphagia was the most common complaint (four cases); while in one case, the problem was solved after endoscopic dilatation. In three cases, bloat and postprandial pain were documented. In one case, an explantation of the mesh was necessary due to mesh migration and painful adhesions. In one further case with gastroparesis, pyloroplasty was performed without success. The data are compared to the available literature. It was found that dysphagia and recurrence rates are unrelated both in biological and in synthetic meshes if the esophagus is encircled. In series preserving the esophagus at least partially uncoated, recurrences after the use of biological meshes relieve dysphagia. After the application of synthetic meshes, dysphagia is aggravated by recurrences.

**Conclusion:**

Recurrence is rare after encircling hiatal hernia repair with the biological mesh Permacol^®^. Dysphagia, gas bloat, and intra-abdominal pain are frequent complaints. Despite the small number of patients, it can be concluded that a biological mesh may be an alternative to synthetic meshes to reduce recurrences at least for up to 2 years. Our study demonstrates that local fibrosis and thickening of the mesh can affect the outcome being associated with abdominal discomfort despite a successful repair. The review of the literature indicates comparable results after 2 years with both biologic and synthetic meshes embracing the esophagus. At the same point in time, reconstruction with synthetic and biologic materials differs when the esophagus is not or only partially encircled in the repair. This is important since encircling artificial meshes can erode the esophagus after 5–10 years.

## Introduction

Surgery for hiatal hernia has gone through many developmental stages after the first repair was reported by Soresi in 1926 (1). The therapy of a large hiatal hernia is far from being established due to the complexity of the anatomical region and the need for improvement of some current methods. In analogy to hernia repair of the abdominal wall, synthetic materials were used to repair hiatal hernia to reduce the risk for hernia recurrence (2). Despite the lower recurrence rates in comparison to direct suture (3), there were significant long-term complications due to local fibrosis, stricture formation around the prosthetic material, esophageal erosion, mesh migration, and late dysphagia (4). To solve these problems, biological meshes from human acellular cadaveric dermis (HACD), porcine small intestine submucosa (SIS), porcine dermal collagen (PDC), or bovine pericardium were developed. HACD and SIS have been utilized as mesh grafts for hiatal hernia repair (5). It is assumed that the natural tissue texture of the biologic mesh results in less esophageal erosions and lowers the risk of complications. Less inflammation and reduced fibrotic tissue changes at the hiatus should lead to a better quality of life, specifically lower dysphagia rates (6).

This study aims to assess the clinical result of patients with large hiatal hernia after repair with a biologic mesh.

## Patients and Methods

A consecutive number of patients, who were diagnosed with large hiatal hernia and thoracic stomach, underwent surgery between 2010 and 2014. Pre- and postoperative work-up included symptoms assessment, barium swallow, endosopy, and CT scan. The large hiatal hernias were anatomically classified as types III and IV and clinically as type 2dII according to Koch et al. (7). Using the formula given by Granderath (8), the hiatal surface area was calculated intraoperatively as 13.5 ± 4.5 cm^2^, which is well in the range of large mixed-type hiatal hernia (9).

**Figure 1:**
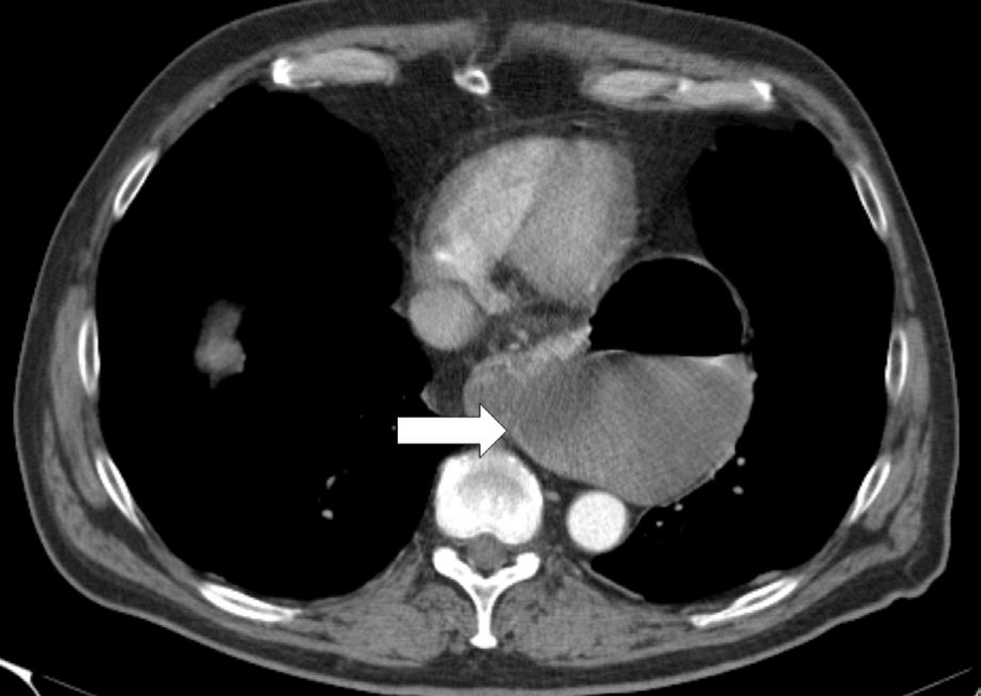
**Preoperative computed tomography of the upside-down stomach of a 73-year-old male with the gastroesophageal junction being fully dislocated into the thoracic cavity (arrow)**.

**Figure 2:**
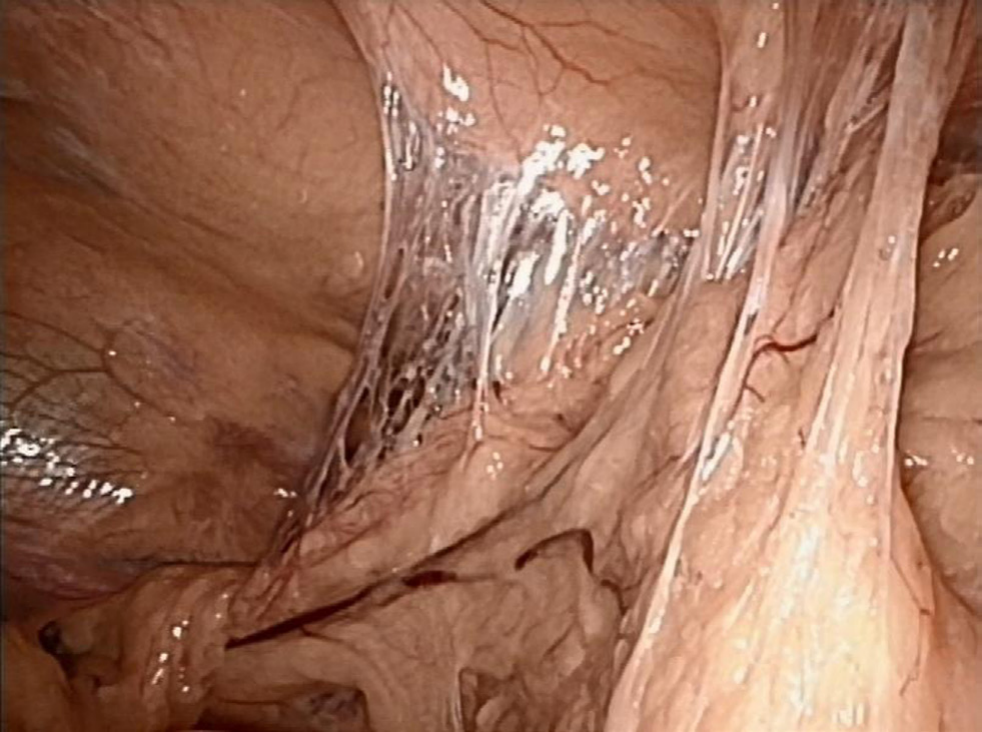
**Situs of a large hiatal hernia in this 72-year-old male operated on 2 years ago**. The upside-down stomach is fully encased in both loose and dense adhesions.

The CT scan in Figure 1 and the intraoperative picture in Figure 2 demonstrate a representative finding of a large hiatal hernia with an upside-down stomach.

Surgery was conducted in the Asklepios Klinikum Harburg in Hamburg, a teaching hospital of the University of Hamburg, and the Asklepios Medical School. All patients complained of unbearable mass reflux with regurgitation of acid material preoperatively. Eight patients complained of the inability to sustain their weight due to dysphagia. Five patients were subsequently unable to conduct routine daily life, such as gardening, wiping of the floor, cycling, etc. One patient suffered from chronic obstructive airway disease and another patient from recurrent pneumonia due to silent aspiration. All hernias were surgically treated by a sutured hiatal repair reinforced with a cross-linked biologic mesh of a porcine acellular dermal collagen matrix (Permacol^®^, Covidien) under general anesthesia. The mesh was placed to circularly enclose the esophagus with an opening of at least 15 mm in diameter. All patients were included prospectively into an inhospital registry. Data including demographics, prior history, and individualized surgical technique were obtained from the patients’ charts and surgical reports. The postoperative progress, the patients’ complaints, and the recurrence status were recorded at regular intervals of maximally 1 year. All case notes were reviewed to determine follow-up and to check specifically whether a recurrence occurred or any further unplanned surgery or endoscopy was required. The current status to date was supplemented by a telephone interview.

### Surgical Procedures

**Figure 3:**
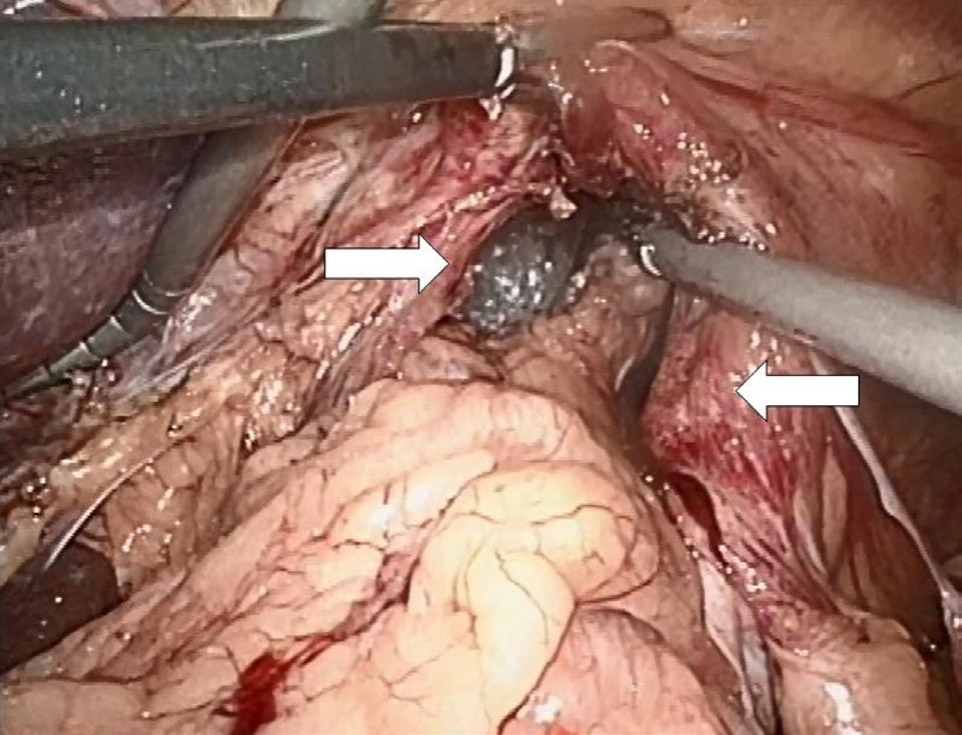
**Preparation of the hiatal sac with the right and the left crus of the diaphragm prepared in their ventral aspect (arrows)**.

**Figure 4:**
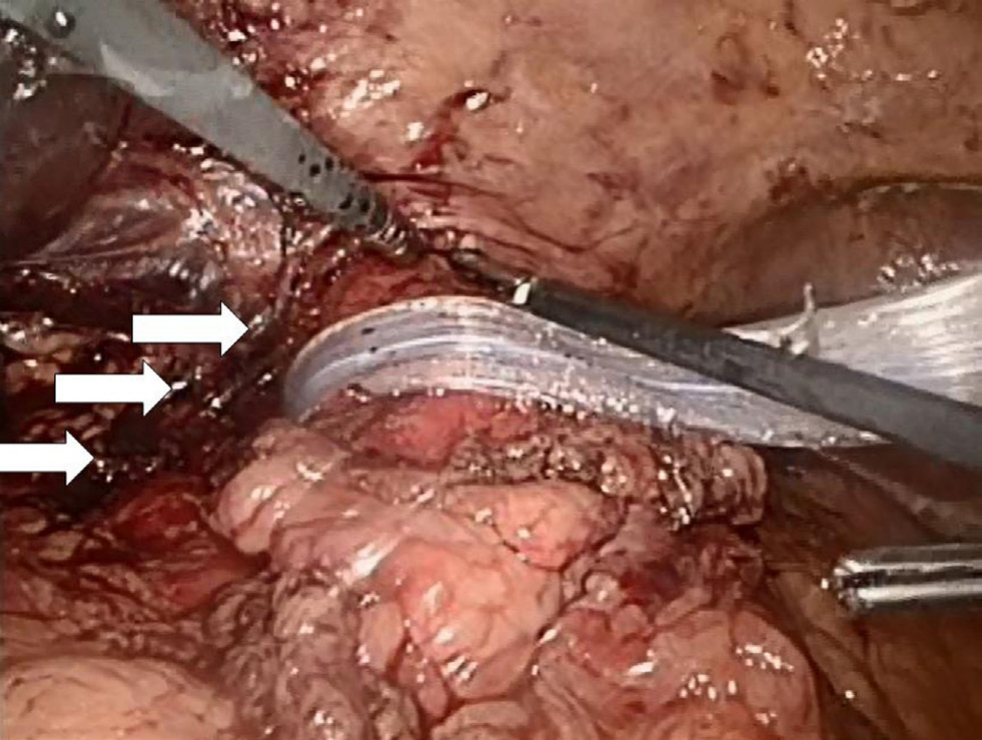
**A hiatoplasty is formed with three evenly spaced non-absorbable sutures (Ethibond 0, Ethicon, Norderstedt, Germany, arrows)**. The distal esophagus is encircled with a silastic band and held to the left. At this point of time, a 54-Ch Rüsch tube is passed through the esophagogastric junction and a 5-mm instrument is additionally placed from the left into the newly formed hiatus in order to ensure sufficient space for the passage of food. A similar instrument is placed at the low left corner of the picture for comparison of sizes. Another 5-mm instrument is inserted from the right in order to lift the ventral crural junction to facilitate instrumentation.

**Figure 5:**
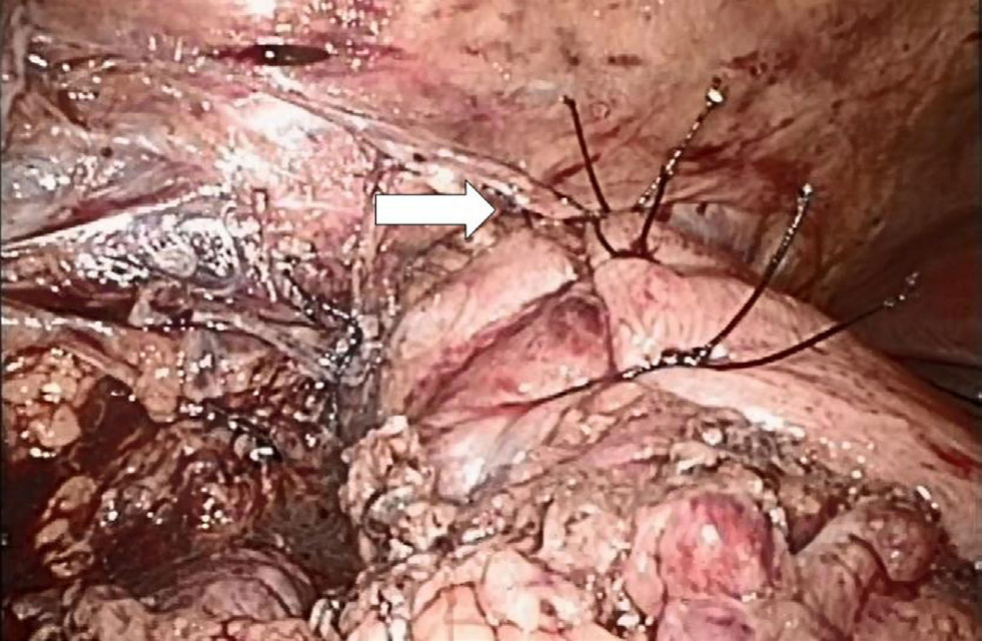
**Short floppy Nissen’s fundoplication in place with the top suture fixing the stomach, esophagus, and right hiatal leg (white arrow)**.

**Figure 6:**
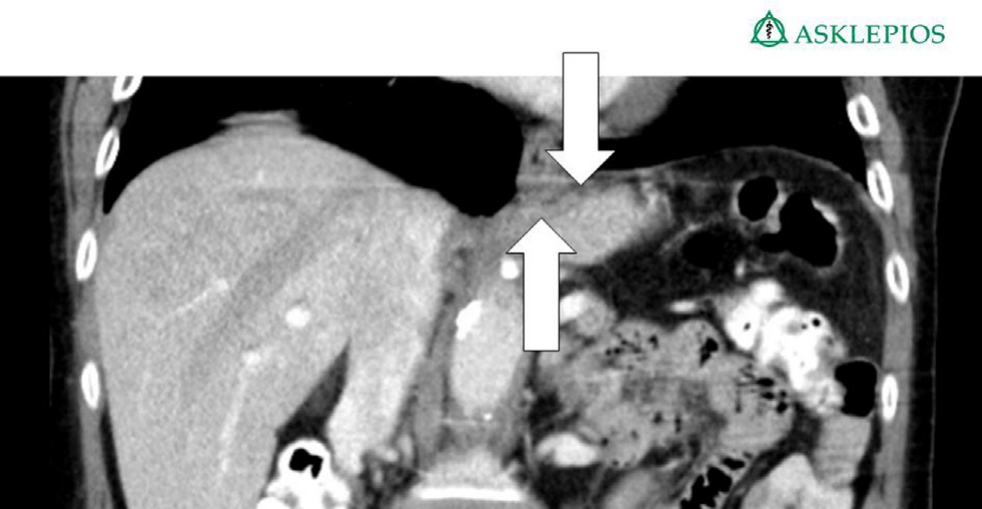
**Hiatal region of a 75-year-old female patient 1 year after implantation of the Permacol^®^ mesh as described above**. The arrows show the position of the mesh. An upper endoscopy showed a mild gastritis without signs of esophageal reflux at this time.

Both laparoscopic and conventional methods were used. The principle was the same: the closure of the paraesophageal hernia using a 10 cm × 10 cm and 1-mm thick biologic mesh of a porcine acellular dermal collagen matrix (Permacol^®^ and Covidien). The surgical technique has previously been described (10). Briefly, the hiatal hernia repair involves the preparation and resection of the sac and reduction and retention of the hernia contents intra-abdominally (Figure 3). The reposition of the stomach back to the abdominal cavity was followed by a wide mediastinal mobilization of the distal esophagus to ensure appropriate intra-abdominal length to prevent the stomach from sliding back up. Retention was achieved with a posterior hiatoplasty (Figure 4) using non-absorbable sutures (Ethibond 0, Ethicon, Norderstedt, Germany). Additionally, a short floppy Nissen’s fundoplication was executed and sutured in place again using Ethibond 0 as three interrupted stitches encircling the intra-abdominal esophagus for 25 mm (Figure 5). The closure of the hiatus was supported with the quadratic biologic mesh. The mesh was tailored to the individual anatomy by rounding the edges, placed on the diaphragmal crura from the abdominal side, sutured into place with at least four non-absorbable sutures (Ethibond 0), and reinforced with fibrin sealant (Evicel^®^, Ethicon^®^, Norderstedt, Germany) (Figure 6).

## Results

During the study period, 13 patients were surgically treated for a thoracic stomach. Among them one patient passed away in the meantime from coronary heart disease. Additionally, two patients were lost to follow-up by moving to an unknown destination – they could not be located either by searching their medical records or by contacting their primary care physicians, leaving a total of 10 patients for further study. There were three (30%) men and seven (70%) women with a median age of 73 ± 13 years (range: 26–81).

**Figure 7:**
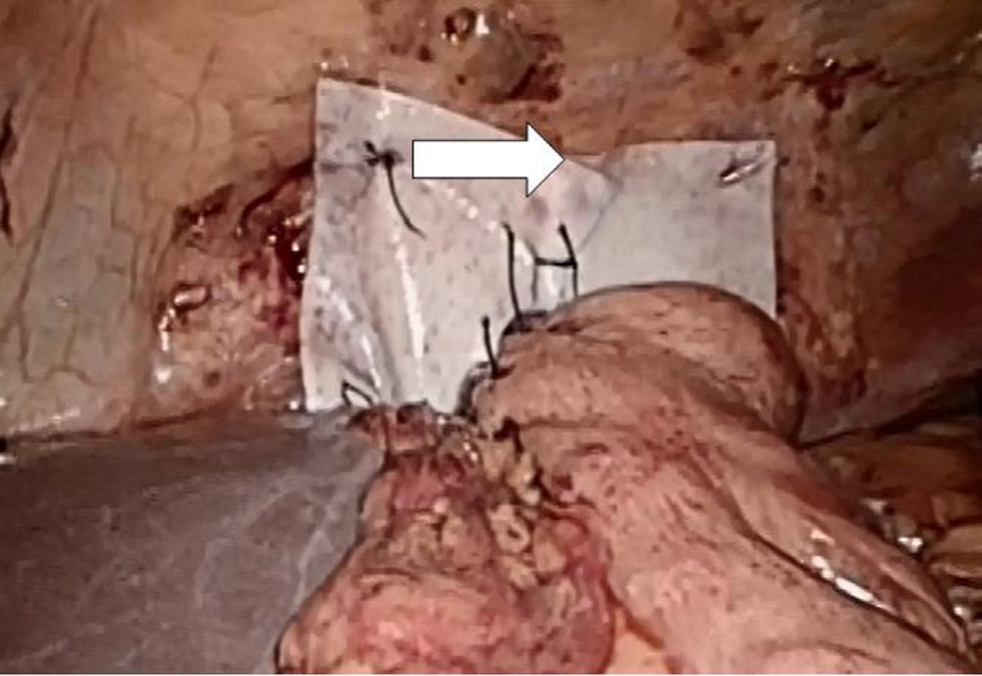
**Permacol^®^ reinforment of the hiatoplasty shown above**. The Permacol^®^ is secured with non-absorbable interrupted stitches and additionally fastened with fibrin glue (Evicell^®^) in critical areas (white arrow).

The mean follow-up was 27 ± 18 months (range: 3–58). The patients underwent in four cases an open procedure twice due to respiratory and once due to cardiocirculatory instability upon laparoscopy. In one case, the laparoscopic approach had to be converted to a combined laparotomy and left-sided thoracotomy due to the inability to reduce the completely intrathoracic stomach in the abdominal cavity. In six cases, a laparoscopic procedure was performed as described above. A total of 20% (2 of 10) underwent one further, unplanned surgery after the prior therapy; in one case, the mesh was explantated because of dysphagia and pain due to dense fibrosis surrounding the mesh. In the other case, pain due to peritoneal adhesions was found unrelated to the sufficient hiatoplasty with mesh enforcement. In the latter case, first, a gastritis was found on repeat gastrocopy, and later, a Herpes zoster infection was elucidated. On an intercurrent computed tomography scan, the mesh was found in place but appears thickened (arrows in Figure 7 below). Measuring the mesh from the scans, a 5-mm plate resulted from the 1-mm mesh in this case.

In this case, a pyloroplasty was performed because of gastroparesis without complete relief of symptoms. Since the patient can keep a normal weight, she is reluctant to any further surgical treatment. There were no other major complications. A total of six patients underwent upper endoscopy because of various complaints, such as burning, intra-abdominal pain, regurgitation, and dysphagia. Except for one case with a stenosis bettered by balloon dilatation and the one reported with a small recurrence, there were no pathological findings related to the hiatal repair.

Four patients (40%) complained about dysphagia postoperatively. In one case, the symptoms declined spontaneously within 6 months. Another patient successfully underwent esophageal dilatation of a stenosis 2 months postoperatively. Up to this date, two patients (20%) still report mild dysphagia but maintain normal body weight. Four cases report persistent intra-abdominal discomfort without weight loss. Three patients experienced bloating, while another patient reported reflux. In another case, regurgitation was described by the patient without abnormal endoscopic findings. The symptoms are summarized in Table 1.

**Table 1:** **Summary of the status after 2 years (multiple declarations possible)**.

Symptom	Cases% (*n*)
Pain	30 (3)
Bloating	30 (3)
Dysphagia	20 (2)
Gastroparesis	10 (1)
Reflux with recurrence	10 (1)
Regurgitation	10 (1)

## Discussion

The treatment of large and giant hiatal hernias has been a challenge, since it is both technically more difficult and has always substantially elevated recurrence rates (10–12). Laparoscopic hiatal hernia repair for larger hernias seem to have even higher recurrence rates leading to revision surgery (13). Primary suture hiatoplasty without reinforcement is associated with high recurrence rates, so that a variety of meshes has been developed to reduce the risk of recurrence (11). The main concern using a synthetic mesh is the risk of specific complications through the local erosion into the stomach, fibrosis, mesh contraction, and esophageal stenosis, which are thought to cause higher dysphagia rates (4, 14–19). Synthetic meshes are associated with a higher risk of esophageal resection at revision surgery (4, 20). In order to minimize these side effects, biologic meshes from human cadaveric dermis and SIS have been developed. A mild inflammatory response and neovascularization were reported for the biologic grafts (21–23). It is believed that a limited foreign body reaction at the hiatus due to their biocompatibility minimizes the risk of postoperative dysphagia (24).

Among the US surgeons who use mesh to repair the hiatal hernia, 67% prefer biologic mesh (25). The recurrence rates following a biological mesh hiatoplasty can vary in the literature between 0 and 54%, with a median value of 10% (Table 2). These recurrence rates are almost identical to those found after hiatal repair using synthetic meshes (range: 0–35, median 7%). In this study, large hiatal hernias were repaired using a cross-linked collagen matrix derived from pig tissue.

**Table 2:** **Pre- and postoperative data from published hiatal repairs embracing the esophagus and reinforcing only the crural repair**.

Author	Year	Mesh type	Patients	Hernia type	Dysphagia preoperatively (%)	Follow-up (months)	Recurrence (%)	Dysphagia 1–2 years postoperatively (%)
**Mesh placement encircling the esophagus**								
Hazebroek (26)	2008	TiMesh	18	II–IV	22	24	6	41
Carlson (27)	1999	PTFE	15	III–IV	na	30	0	na
Frantzides (28)	2002	PTFE	36	III–IV	na	30	0	na
Lubezky (29)	2007	PTFE/ePTFE	45	III–IV	17	28	13	20
Stavropoulos (30)	2012	ePTFE	38	II–IV	na	24	na	na
Zaninotto (31)	2007	Polypropylene/ePTFE	35	III	na	71	9	22
Gouvas (32)	2011	Polypropylene/PTFE	20	II–IV	84	36	15	19
Chilintseva (33)	2012	PTFE/polyester/polypropylene/ePTFE	45	I–IV	7	51	4	11
Oelschläger (34)	2003	SIS	9	II–III	33	8	0	13
Jacobs (35)	2007	SIS	92	I–III	na	38	3	11
Massullo (36)	2012	Polyglycolic:trimethylene	11	I–III	na	13	9	0
Present paper	2015	Cross-linked acellular pig dermis	10	III–IV	80	26	10	20
**Mesh placement avoiding the esophagus**								
Watson (37)	2015	TiMesh	42	III–IV	19	12	23	7
Gryska (38)	2005	PTFE	130	I–III	na	48	8	0
Hazebroek (26)	2009	ePTFE	14	II–III	16	34	29	27
Champion (39)	2003	Polypropylene	19	II–III	na	25	5	11
Leeder (40)	2003	Polypropylene	14	I–III	93	46	14	0
Horstmann (41)	2004	Polypropylene	16	II–III	31	14	0	na
Granderath (42)	2006	Polypropylene	150	II–IV	na	12	8	4
Turkcapar (43)	2007	Polypropylene	156	I–II	na	24	2	1
Soricelli (44)	2009	Polypropylene	91	II–III	na	69	2	0
Morino (45)	2006	Polypropylene/PTFE	37	I–III	na	36	35	0
Grubnik (46)	2013	Polypropylene-Monocryl	158	II–IV	na	28	5	2
Goers (47)	2011	Various biomeshes	40	II–IV	na	6	0	38
Molena (48)	2015	Various biomeshes	18	III–IV	na	na	na	na
Ringley (49)	2006	HACD	22	II–IV	0	7	0	6
Wisbach (50)	2006	HACD	11	III	55	24	11	18
Lee (51)	2007	HACD	17	I–III	na	14	12	6
Lee (52)	2008	HACD	52	na	44	24	4	na
Diaz (53)	2011	HACD	26	II–III	13	24	15	23
Alicuben (54)	2014	HACD	15	II–IV	na	12	20	13
**Mesh placement avoiding the esophagus**								
Jacobs (35)	2007	SIS	74	na	na	38	4	na
Fumagalli (55)	2008	SIS	6	na	na	12	50	13
Oelschlager (56)	2011	SIS	33	II–III	3	58	54	3
Wassenaar (57)	2012	SIS	31	I–IV	na	45	3	20
Watson (37)	2015	SIS	41	III–IV	27	12	23	9
Wang (58)	2015	SIS and alike	66	I–III	6	24	13	4

*na, not available*.

According to a retrospective analysis of hiatal revisions following synthetic or biologic mesh application, there were no significant differences in terms of blood loss, duration of surgery, morbidity, and need for esophageal reconstruction (17). The recurrence rate can be significantly reduced from 16 to 0% with the use of an absorbable mesh for the repair of small hiatal hernia (23). In a study of 108 patients, the recurrence rate was reduced from 24 to 9% with the laparoscopic use of a biologic mesh compared to the suture of the hiatus (7). A new meta-analysis confirmed the lower recurrence rates for the biologic mesh in the short-term, but the long-term benefit remains unclear (24). The repair of large hiatal hernias with biologic mesh may be associated with a lower risk for short-term recurrence compared to primary suture repair. Short-term recurrence rates for suture repair and biologic mesh repair ranged in a meta-analysis between 16.6 and 3.5%, respectively. The same study showed that the long-term recurrence based on data provided by one trial only was 51.3 and 42.4%, respectively (25). In our study, the recurrence rate was 10% after a median time of 27 months. The recurrence rate after the use of SIS can be up to 9% (26). Another large study with 92 patients treated with SIS achieved a recurrence rate of 3.3% and a dysphagia rate of 8.6% in a median follow-up of 3.3 years (27). The incidence of postoperative dysphagia in 22 patients after treatment with human acellular dermal matrix was 4.5% (28). In our study, 40% of the patients complaint postoperatively about dysphagia. Up to now and after successful endoscopic dilatation in one case, 20% of the patients still have the sensation of dysphagia but keep their weight.

**Figure 8:**
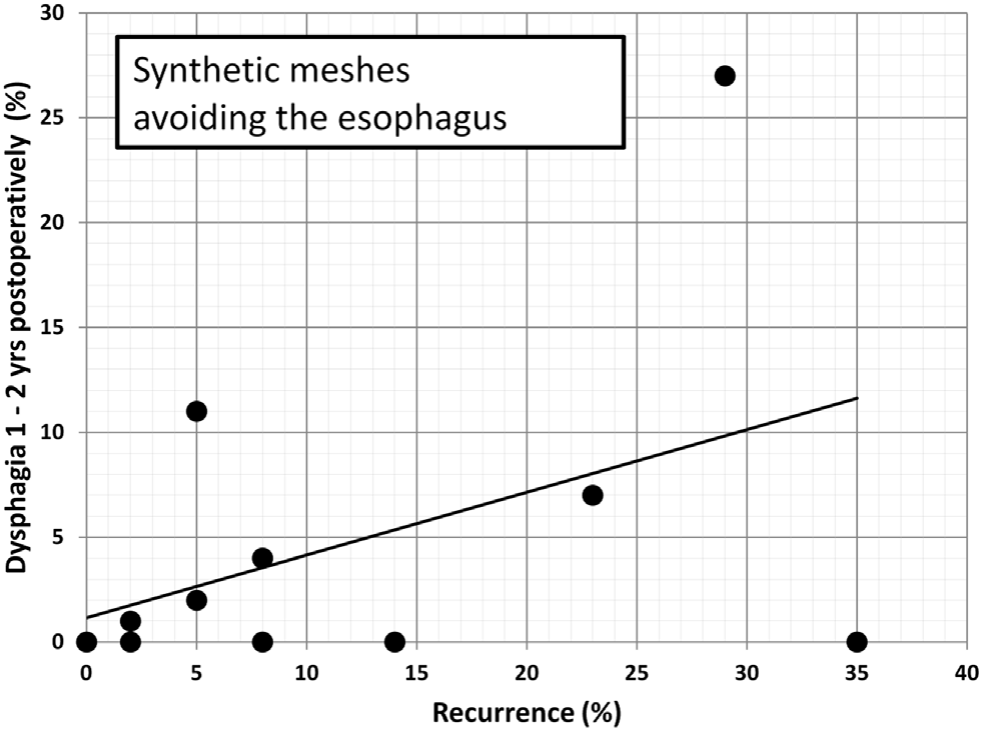
**Dysphagia rates as a function of recurring hiatal hernia after 1–2 years using synthetic meshes leaving at least half of the circumference of the esophagus to move freely (data from Table 2)**. The line indicates the trendline.

**Figure 9:**
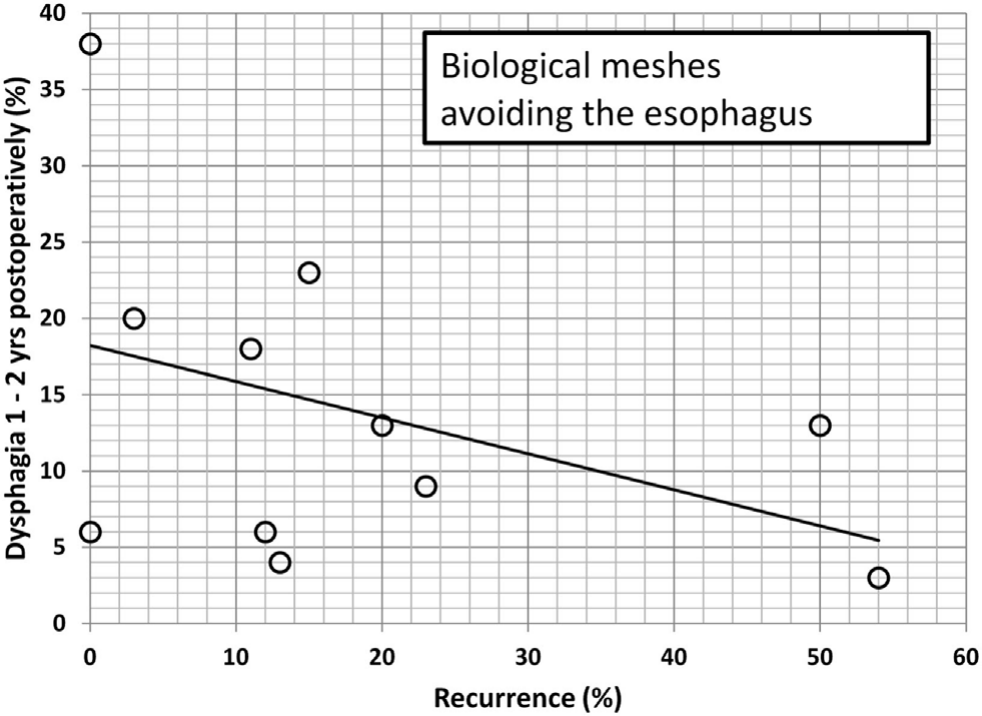
**Dysphagia rates as a function of recurring hiatal hernia after 1–2 years using biological meshes leaving at least half of the circumference of the esophagus to move freely (data from Table 2)**. The line indicates the trendline.

Since dysphagia impairs the quality of life significantly, an attempt is made to further elucidate potential associations. In Table 2, data are accumulated from the available literature attempting an assessment at a certain postoperative period, namely, 1–2 years as observed in this manuscript. The data are divided in biological and synthetic meshes using techniques embracing the esophagus in order to reduced long-term recurrence rate or excluding the esophagus in an attempt to preserve its function. Since it cannot be assumed that the data are homogenously distributed, the Mann–Whitney *U*-test was used to evaluate group differences. Neither the preoperative dysphagia rate nor the placement of the mesh influences the dysphagia rate significantly although meshes encircling the esophagus tend to exhibit higher dysphagia rates (Table 2, *p* = 0.126). The length of the follow-up and the rate of recurrence or dysphagia are unrelated in all groups (Table 2, *p* = 0.667). In both synthetic and biological meshes embracing the esophagus, there is a trend toward elevated dysphagia rates with increasing recurrence rates (Table 2, *p* = 0.021). In reconstructions avoiding at least half of the circumference, synthetic meshes increase dysphagia as recurrences occur [Table 2; Figure 8, *r* = 0.63, small effect according to Thalheimer and Cook (59)]. In contrast, biological meshes decrease dysphagia rates as recurrences occur [Table 2; Figure 9, *r* = −0.728, intermediate effect according to Thalheimer and Cook (59)]. The results should be viewed with caution but can be interpreted that the integration of biological meshes in reconstructions avoiding the esophagus decreases dysphagia increasing recurrences within the first 2 years.

In a recent study with 49 patients, 8% required endoscopic dilatation with a successful resolution of the symptoms (60). Interestingly, hiatal hernia repair with biomesh fails to increase the postoperative dysphagia rate compared to suture repair alone (6). The reported rates of dysphagia with synthetic meshes vary between 0 and 41%, with a median of 15.5% [Table 2 (24, 61–63)].

Most patients with a recurrent paraesophageal hernia still experience an improvement of clinical symptoms compared to the preoperative status (63, 64). Despite the high recurrence rate up to 54% after a laparoscopic repair of the hiatus with or without mesh, there can be a significant improvement in all parameters assessing the quality of life (10). This can be due to the smaller sac of the recurrent hernia compared to the original size with a diminished risk of volvulus, obstruction, and ischemia. Our findings demonstrate, in general, that gastrointestinal symptoms associated with big paraesophageal hernias and thoracic stomach, such as postprandial obstruction and pain, are significantly improved after a mesh repair up to 58 months following the surgical closure and reinforcement with the biomesh Permacol^®^. However, price and limited use, e.g., for religious reasons should be weighed against the potential of the biomesh.

Cross-linked collagen matrices have a more coordinated structure and therefore can sustain higher loads for longer times compared to non-cross-linked ones (60, 65). Several studies showed that cross-linking does not appear to affect the tissue integration in animal models or human (60, 65, 66). Mesh fibrosis may occur potentially increasing the stiffness of the repair (as demonstrated in a postoperative CT scan, Figure 7). Late onset dysphagia even in mesh positions avoiding the esophagus might be related to this scar formation (60). It remains unclear whether cross-linking contributes to fibrotic changes since we know that resorption is delayed. Permacol™ is a porcine-derived acellular dermal sheet, which is composed predominantly of type I collagen (93–95%). During the manufacturing process, the cellular components are removed and the collagen of the dermis is treated with hexamethylene diisocyanate (HMDI) to increase the degree of cross-linking. It is currently used for the repair of abdominal and thoracic wall defects and for hernias (60). To prevent mesh dislocation, meshes must be fixated (67). So far, little is known how to best fasten a hernia mesh in the hiatal position. There are many different ways to anchor a mesh, such as non-absorbable sutures, tacks, or fibrin sealant (61). We prefer a limited number of sutures and add fibrin glue as shown in Figure 6 in order to achieve a maximal pliability still holding the mesh in place at the same time. Since the mesh can be placed in at least six different positions, the best placement is still unknown. Most surgeons place a mesh in a U-shape or a pantaloon collar in a retroesophageal position with the limbs of the mesh encircling the esophagus [Table 2 (10–64)]. Data depicted in Figures 8 and 9 indicate a different behavior of synthetic and biologic meshes when the esophagus is not fully encircled. On the one hand, patients with smaller (up to 5 cm) hiatal hernias may benefit from the use of a biologic mesh for the repair (61, 64, 68). On the other hand, larger hernias are more prone to develop recurrences, even with mesh reinforcement (68). At this point of time, the preferred technique, the superior mesh position, or the outstanding material still awaits future investigation.

## Conclusion

The principle of hiatal hernia repair aims to eliminate the hernia preserving the functionality of the gastroesophageal junction at the same time. The use of a biologic mesh to repair large hiatal hernias is an effective method with low recurrence rates. It can reduce the local inflammation and postoperative dysphagia compared to synthetic meshes. Our study demonstrates that local fibrosis and thickening of the mesh can affect the outcome being associated with abdominal discomfort despite a successful repair. The review of the literature indicates comparable results after 2 years with both biologic and synthetic meshes embracing the esophagus. At the same point of time, reconstruction with synthetic and biologic materials differs when the esophagus is not or only partially encircled in the repair.

## Ethics Statement

Retrospective case series. No ethic committee approval necessary.

## Author Contributions

FA and FK have treated the patients in their hospital. The development of the study design and the follow-up of the patients have been also done by FA and FK. FA, FKö, and FK are responsible for the content of the manuscript.

## Conflict of Interest Statement

The authors declare that the research was conducted in the absence of any commercial or financial relationships that could be construed as a potential conflict of interest.
